# Soft x-ray absorption spectroscopy of metalloproteins and high-valent metal-complexes at room temperature using free-electron lasers

**DOI:** 10.1063/1.4986627

**Published:** 2017-09-01

**Authors:** Markus Kubin, Jan Kern, Sheraz Gul, Thomas Kroll, Ruchira Chatterjee, Heike Löchel, Franklin D. Fuller, Raymond G. Sierra, Wilson Quevedo, Christian Weniger, Jens Rehanek, Anatoly Firsov, Hartawan Laksmono, Clemens Weninger, Roberto Alonso-Mori, Dennis L. Nordlund, Benedikt Lassalle-Kaiser, James M. Glownia, Jacek Krzywinski, Stefan Moeller, Joshua J. Turner, Michael P. Minitti, Georgi L. Dakovski, Sergey Koroidov, Anurag Kawde, Jacob S. Kanady, Emily Y. Tsui, Sandy Suseno, Zhiji Han, Ethan Hill, Taketo Taguchi, Andrew S. Borovik, Theodor Agapie, Johannes Messinger, Alexei Erko, Alexander Föhlisch, Uwe Bergmann, Rolf Mitzner, Vittal K. Yachandra, Junko Yano, Philippe Wernet

**Affiliations:** 1 Institute for Methods and Instrumentation for Synchrotron Radiation Research, Helmholtz-Zentrum Berlin für Materialien und Energie GmbH, 12489 Berlin, Germany; 2 Molecular Biophysics and Integrated Bioimaging Division, Lawrence Berkeley National Laboratory, Berkeley, California 94720, USA; 3 Linac Coherent Light Source, SLAC National Accelerator Laboratory, Menlo Park, California 94025, USA; 4 Stanford Synchrotron Radiation Lightsource, SLAC National Accelerator Laboratory, Menlo Park, California 94025, USA; 5 Institute for Nanometre Optics and Technology, Helmholtz-Zentrum Berlin für Materialien und Energie GmbH, 12489 Berlin, Germany; 6 Stanford PULSE Institute, SLAC National Accelerator Laboratory, Menlo Park, California 94025, USA; 7 Synchrotron SOLEIL, L'Orme des Merisiers, Saint-Aubin, 91191 Gif-sur-Yvette, France; 8 Institutionen för Kemi, Kemiskt Biologiskt Centrum, Umeå Universitet, SE 90187 Umeå, Sweden; 9 Division of Chemistry and Chemical Engineering, California Institute of Technology, Pasadena, California 91125, USA; 10 Department of Chemistry, University of California-Irvine, 1102 Natural Sciences II, Irvine, California 92697-2025, USA; 11 Department of Chemistry, Molecular Biomimetics, Ångström Laboratory, Uppsala University, SE 75237 Uppsala, Sweden; 12 Institut für Physik und Astronomie, Universität Potsdam, 14476 Potsdam, Germany

## Abstract

X-ray absorption spectroscopy at the L-edge of 3d transition metals provides unique information on the local metal charge and spin states by directly probing 3d-derived molecular orbitals through 2p-3d transitions. However, this soft x-ray technique has been rarely used at synchrotron facilities for mechanistic studies of metalloenzymes due to the difficulties of x-ray-induced sample damage and strong background signals from light elements that can dominate the low metal signal. Here, we combine femtosecond soft x-ray pulses from a free-electron laser with a novel x-ray fluorescence-yield spectrometer to overcome these difficulties. We present L-edge absorption spectra of inorganic high-valent Mn complexes (Mn ∼ 6–15 mmol/l) with no visible effects of radiation damage. We also present the first L-edge absorption spectra of the oxygen evolving complex (Mn_4_CaO_5_) in Photosystem II (Mn < 1 mmol/l) at room temperature, measured under similar conditions. Our approach opens new ways to study metalloenzymes under functional conditions.

## I. INTRODUCTION

Many important redox-active metalloenzymes such as Photosystem II (PS II), hydrogenases, and nitrogenases employ 3d transition metals in their active sites, where they catalyze multi-electron reactions in aqueous solution, at ambient temperature and pressure.^1–3^ While these catalysts cannot simply be transferred into industrial processes, they provide unique information on how to spatially and temporally control electron and proton flow and product/substrate transport during chemical transformations.

To probe the chemistry of such biological and related inorganic catalytic sites, metal K-edge spectroscopy (1s to 3d and np transitions) in the hard x-ray energy range has been widely used, providing element-specific information on the electronic structure and the local coordination environment of the metals.^4–9^ In contrast, metal L-edge spectroscopy, which probes 2p → 3d transitions, has been rarely applied to biological systems despite several advantages. These transitions are dipole-allowed, show greater sensitivity to the occupancy, spin state, and ligand interactions of the metal 3d derived orbitals,^10–12^ and exhibit a smaller inherent spectral broadening (due to longer core-hole lifetime), as compared to the metal K-edge.^13^ In fact, the field of materials science has recognized these advantages, and L-edge spectroscopy of 3d transition metals has provided important electronic structural information through x-ray absorption (XAS) and emission spectroscopy (XES), as well as 2p → 3d resonant inelastic x-ray scattering spectroscopy (RIXS).^12,14–18^ This difference between spectroscopy on materials and on biological catalytic sites arises from several factors: (i) X-ray induced sample damage strongly limits spectroscopic information at soft x-ray energies, even at cryogenic temperatures.^19–21^ (ii) Biological metalloprotein solution samples are comparably dilute and have metal concentrations mostly on the order of 1 mmol/l (1 mM), which poses experimental challenges for discriminating the signal of the probed metal center over that of the dominant background due to absorption and fluorescence signals by light elements such as C, N, and O in the protein and in the solvent.^16,22^ (iii) Soft x-rays strongly interact with matter and hence require a vacuum environment, which dehydrates the samples and prevents catalytic turnover.^23^

In recent years, the development of x-ray free-electron laser (XFEL) sources has provided x-ray pulses with high brilliance and durations in the femtosecond (fs) domain.^24,25^ This has enabled the fast-emerging field of x-ray diffraction and x-ray spectroscopy of proteins in the hard x-ray energy range under biologically functional conditions, while overcoming the limits set by x-ray induced sample damage.^7,26–30^ In a similar manner, biological soft x-ray spectroscopy can take advantage of XFELs to collect x-ray damage-free data at room temperature by outrunning the sample damage with fs pulses if a suitable detection scheme is used. Such a detection scheme needs to extract the L_α,β_ fluorescence signal (400–1000 eV) arising from the dilute metal sites and separate it from the very strong background from K_α_ fluorescence (277–525 eV), emitted by light elements in the sample (C, N, and O). This can be realized with an energy discrimination scheme making use of the element-specific partial fluorescence yield (PFY) detection.

**FIG. 1.**
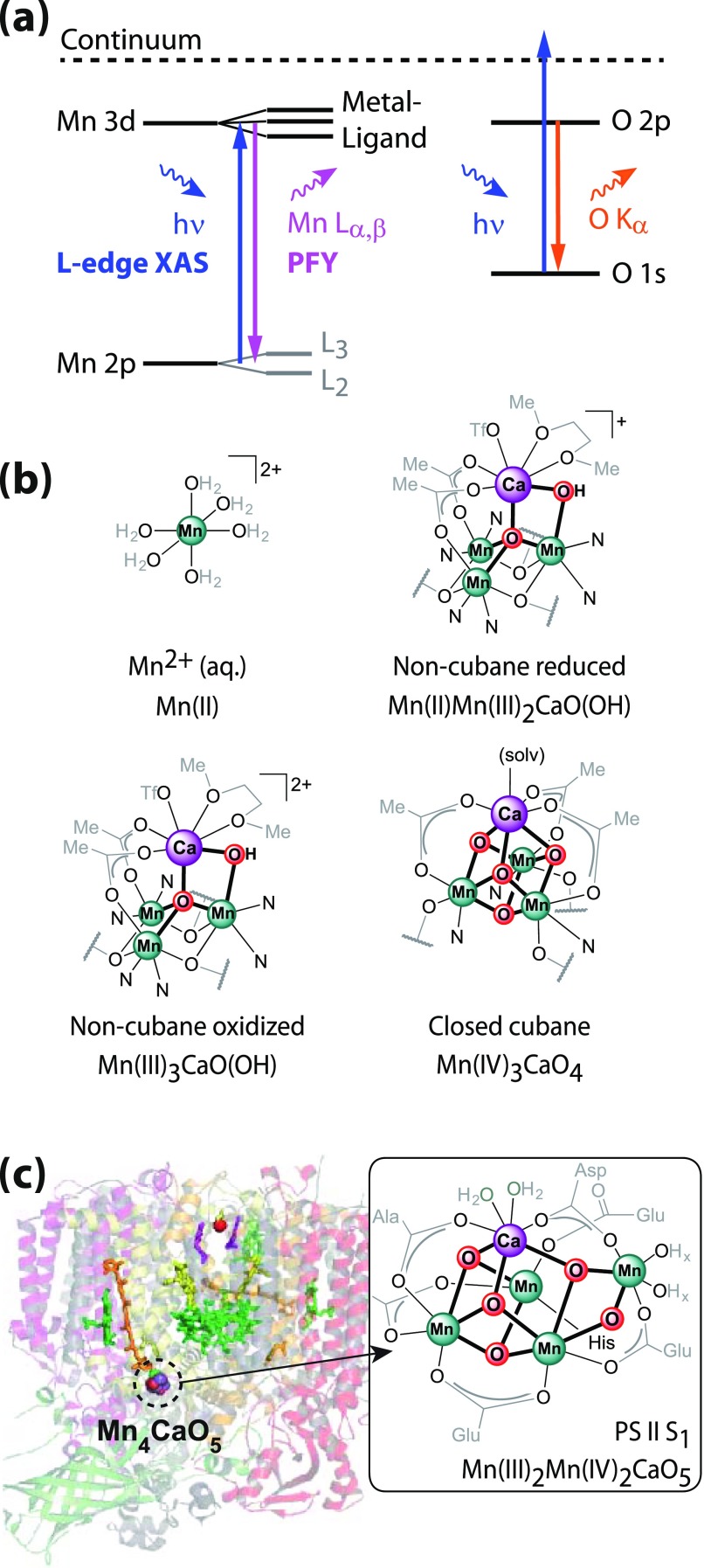
Probing scheme and local structure of the Mn sites investigated in this work with sample names and assigned oxidation states of Mn. (a) Scheme of Mn L-edge absorption spectroscopy with dominating one-electron transitions for absorption and fluorescence at the Mn L and O K-edges. (*left*) Partial fluorescence yield (PFY) x-ray absorption spectroscopy (PFY-XAS) at the Mn L-edge corresponds to detecting the Mn L_α,β_ fluorescence signal (Mn 3d → 2p transitions) as a function of incident photon energy across the Mn L_3,2_ absorption edges (resonant Mn 2p → 3d transitions). Spin-orbit interactions in the Mn 2p shell split the absorption spectrum into L_3_ and L_2_ edges. (*right*) The concurrent O K_α_ fluorescence (O 2p → 1 s transitions) resulting from 1 s ionization of O in the sample (non-resonant O 1 s → continuum transitions) is also indicated. (b) Four inorganic mono- and multinuclear high-spin Mn complexes with variable oxidation states and molecular structures. (c) The photosystem II protein^1^ and the Mn_4_CaO_5_ cluster (inset adapted from Ref. 30 for the protein in the dark resting S_1_ state).

Recently, we introduced a spectrometer for x-ray absorption spectroscopy with partial-fluorescence yield detection (PFY-XAS) based on three reflective zone plates (RZPs).^16^ RZPs have the potential for high photon efficiency due to their simultaneous dispersive and focusing behavior, combined in a single optical element with a large acceptance (solid) angle. The dispersive behavior allows us to record different energies of the emitted fluorescence at different positions on a 2D detector, while the focusing behavior allows the increasing S/N ratio of a selected emission energy and other emission energies are defocused. In the case of PFY-XAS on the Mn L-edge, the RZPs have been optimized to separate the Mn L_α,β_ (3d → 2p) fluorescence at ∼637 eV subsequent to Mn L-edge (2p → 3d) absorption [Fig. 1(a)] from the O K_α_-edge fluorescence at 525 eV with a bandwidth of 20 eV (FWHM).^31^ In a previous proof of principle experiment, using an ionic Mn model system in aqueous solution (Mn concentration, ∼500 mM), we have demonstrated the viability of the concept for collecting PFY-XAS at an XFEL source at physiological temperature and pressure.^16,22^ However, the detection of PFY-XAS signals from 100 to 500 times more dilute metal centers in molecular inorganic catalysts and metalloenzymes has hitherto proven elusive due to the insufficient signal-to-noise (S/N) ratio.

In this study, we report Mn L-edge spectra of high-valent Mn high-spin complexes [Fig. 1(b)] in solution and at room temperature with metal concentrations on the order of 1 to 10 mM. The XFEL based work has become possible with the application of an improved RZP spectrometer to collect the PFY signal from dilute samples. With the example of the water oxidation catalyst (Mn_4_CaO_5_) in PS II solution [Fig. 1(c)], we show that L-edge spectroscopy of dilute metal centers in metalloproteins under functional conditions is now within reach. PS II catalyzes the water oxidation reaction in photosynthesis. Upon sequential absorption of visible photons, it advances in a series of intermediate states S_0_ → S_1_ → S_2_ → S_3_ → S_4_ and accumulates four oxidizing equivalents (unit charges) in the Mn_4_CaO_5_ cluster.^1,32^ We interpret Mn L-edge spectra of the Mn_4_CaO_5_ cluster in PS II in the dark resting state and in an illuminated state by comparing them to experimental spectra of structurally well-characterized Mn model complexes,^33,34^ measured under similar conditions.

## II. RESULTS AND DISCUSSION

### A. PFY-XAS on dilute transition metals using a reflection zone plate spectrometer

**FIG. 2.**
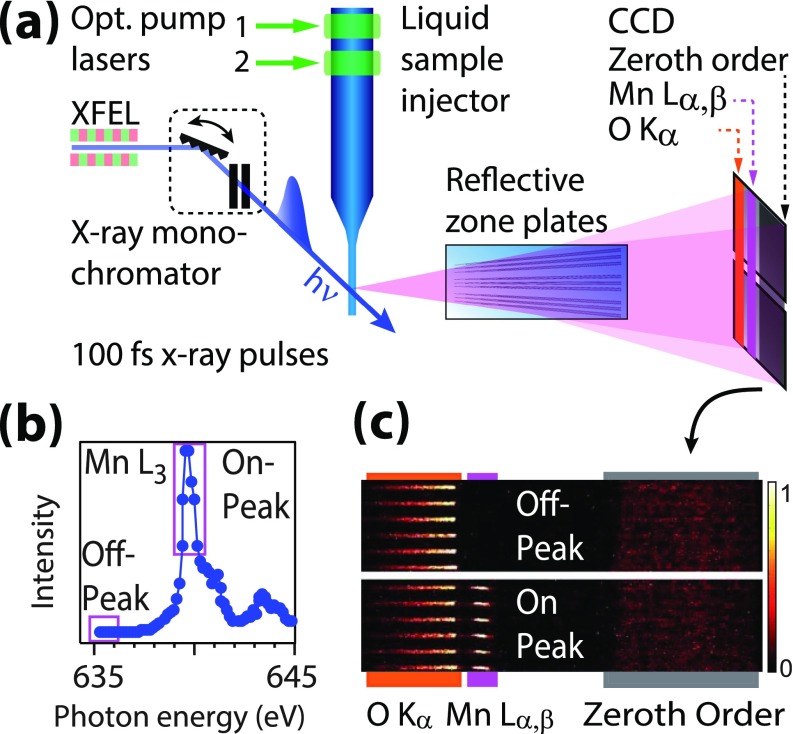
Concept of the experimental design. (a) Setup for Mn L-edge PFY-XAS on dilute samples in solution with optical pump lasers (green arrows, for illumination of the PS II sample) and femtosecond soft x-ray probe pulses (blue arrow) from the Linac Coherent Light Source (LCLS) x-ray free-electron laser (XFEL), probing the liquid sample jet. For PXY-XAS on the Mn L-edge, the incident photon energy is scanned stepwise with the x-ray monochromator of the soft x-ray beamline of the LCLS XFEL. At each step, a reflective zone plate spectrometer separates the Mn L_α,β_ from the O K_α_ fluorescence in the -1st diffraction order (and the total fluorescence signal in the 0th order reflection), which are all simultaneously detected with a CCD camera. (b) Example of a Mn L_3_-edge PFY-XAS spectrum of a 500 mM Mn^2+^_aq_ solution sample, which was obtained with the (normalized) integrated Mn L_α,β_ fluorescence intensity on the CCD image in (c) as a function of the incident photon energy. (c) Comparison of the CCD images averaged over the “On-Peak” and “Off-Peak” data points assigned in (b) with the same color scale (in photons/5 s).

Our focus here is to probe dilute solution samples at room temperature, provided by liquid sample injection systems^35,36^ which avoid dehydration and freezing of the samples in the high-vacuum environment required for soft X-ray spectroscopy. Probing the sample in solution at room temperature is necessary for studying chemical reactions under functional conditions in proteins and many molecular inorganic catalysts. The experimental setup at the Linac Coherent Light Source (LCLS) XFEL (Stanford, USA) combines a liquid jet delivery system with an *in-situ* visible pump and a RZP spectrometer for PFY-XAS detection as shown in Fig. 2(a). We note that using a liquid-sample cell with an x-ray transmissive membrane as in Ref. 37 is not suitable for the high-valent complexes studied here due to their high sensitivity to x-ray damage. For dilute samples in solution with metal concentrations on the order of 1–10 mM, the amount of the fluorescence signal from light elements in the sample solution creates a dominant background signal. In our setup, an array of RZP optics achieves the element-specific detection of the PFY signal and the separation from this background signal. Each RZP element disperses the fluorescence photons by their photon energy in the −1st diffraction order and focuses the PFY of the probed metal center, in our case the Mn L_α,β_ fluorescence, onto the detector plane. The CCD detector simultaneously captures the Mn L_α,β_ signal together with the O K_α_ fluorescence and the total fluorescence yield (TFY) in the 0th order reflection from the RZPs. Compared to our earlier proof-of-principle experiments with a 3 RZP array spectrometer,^16^ we have improved the detection and alignment capabilities, enabling us to record the spectra presented in this paper. The improvements consist of an increased effective size of the RZP array from 3 to 15^22,31^ and now 54 active RZP structures. Furthermore, we increased the solid angle by using two instead of one CCD detector. The photon detection efficiency was increased by ∼1.4 as compared to Ref. 16. Finally, a number of conceptual improvements were realized. These include an improved separation of Mn and O fluorescence on the detector and lithographically made structures on the RZPs for fast and easy alignment of the spectrometer. We note that the concept we describe here is applicable to other 3d transition metals by adapting the optical properties of the RZP structures.

To record PFY-XAS spectra with this setup, we use the beam line monochromator to stepwise select a narrow-bandwidth (0.4–0.6 eV) slice of incident photon energies out of the broad SASE pulses (∼4 eV) provided by the LCLS XFEL. At each step, we integrate the PFY and TFY signals on the CCD image. The normalized PFY signal of Mn as a function of the incident photon energy gives the PFY-XAS spectrum.^16,38^ The TFY signal in the 0th order reflection is essential for an accurate normalization of the PFY intensity of the probed metal, and this signal is directly proportional to the portion of the beamline flux hitting the sample. This approach is particularly important for the low sample concentrations used in this study, where the contribution of Mn fluorescence to the total fluorescence signal is negligible.

An example spectrum of the L_3_ absorption edge of Mn^2+^ in aqueous solution, recorded with this setup at the LCLS XFEL, is shown in Fig. 2(b). Each data point on the y-axis in this figure is the integrated Mn L_α,β_ fluorescence signal in the area assigned on the CCD images in Fig. 2(c). The “Off-peak” (top) and “On-peak” (bottom) panels of this figure show the CCD images averaged over the data points below the absorption onset and in the Mn L_3_ peak region, respectively, as assigned in Fig. 2(b). The “On-peak” (bottom) panel illustrates the spatial separation of the row of the focused Mn L_α,β_ fluorescence spots centered at ∼637 eV from the O K_α_ fluorescence signals at ∼525 eV (stripe shaped signals) and from the flat-top feature originating from the 0th order reflection. For illustration, a representative fraction of CCD signals from 6 of 54 RZPs is shown. The comparison of CCD images, averaged over the “Off-peak” and “On-peak” spectral regions of Mn L_3_, illustrates the concept of PFY-XAS detection: The row of Mn L_α,β_ signal spots changes its intensity as a function of the incident photon energy. The agreement of the Mn L_3_-edge spectrum of a Mn^2+^_aq_ solution sample measured here [Fig. 2(b)] with previously published data measured at a quasi-continuous synchrotron source^22^ demonstrates the validity of our approach at the XFEL source (see also supplementary material).

**FIG. 3.**
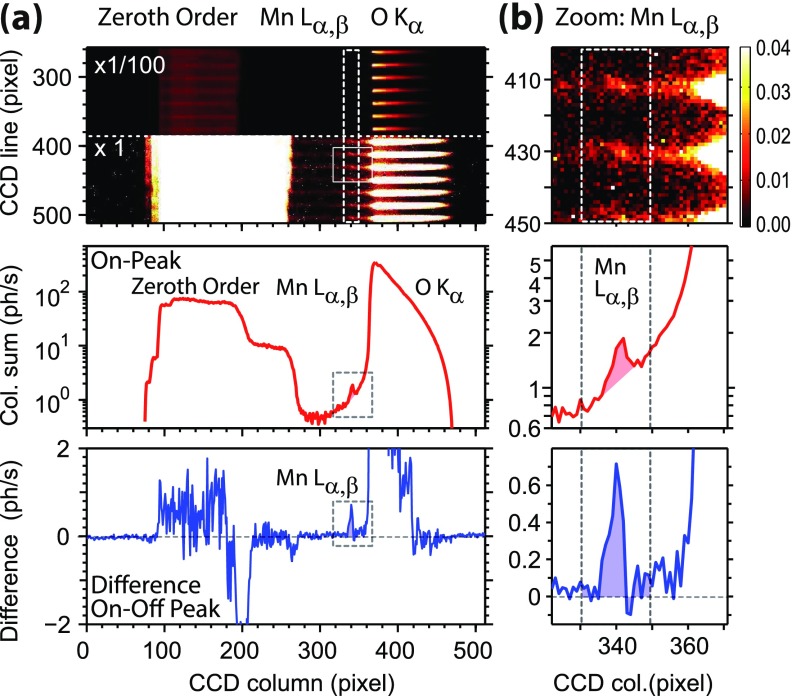
CCD signals of the PS II solution sample at room temperature (Mn concentration, 0.8 mM). (a) (*top*) Mn L_3_ “On-Peak” (639.4 eV < hν< 644.8 eV) average of a single CCD with the color code given in photons/second and (*middle*) the corresponding projection to the x-axis. (*bottom*) Difference in the count rates averaged “On-Peak” minus “Off-Peak” (hν< 637.5 eV). The Mn L_α,β_ fluorescence with ∼3 ph/s (shaded area) is focused to 4 × 4 pixel (220 *μ*m) wide spots, which are clearly separated from the dominant O K_α_ fluorescence. (b) Zoom into the panels of (a) with focus on the Mn L_α,β_ region. Note that two available CCDs recorded a Mn L_α,β_ fluorescence signal of ∼5 ph/s, whereas the signal of one CCD is shown here.

For biological samples, the concentration of the metal centers is often in the range of 1–10 mM. For example, in contrast to the readily distinguishable Mn L_α,β_ fluorescence signal of a highly concentrated Mn^2+^_aq_ complex in Fig. 2(c), the stoichiometric ratio of Mn:O in the PS II solution amounts to 1:64000 with a Mn concentration of 0.8 mM. Our improved RZP spectrometer enables us to separate the weak Mn L_α,β_ fluorescence from the overwhelming O K_α_ fluorescence. Representative experimental detector signals from a PS II solution sample with a Mn concentration of 0.8 mM are shown in Figs. 3(a) and 3(b) (top panels) where panel (b) depicts magnifications of the Mn L_α,β_ fluorescence signal from the overall detector signal shown in panel (a). These images show the signal of one CCD in the spectrometer, averaged over the FWHM spectral range of the measured Mn L_3_-edge (639.4–644.8 eV, On-peak region). The CCD images (top panels) and the sum projections (middle panels) in Fig. 3 exhibit a distinct Mn fluorescence peak with adjacent strong O fluorescence intensity. This demonstrates the spatial or, equivalently, spectral separation of the respective fluorescence signals. The total “On-Peak” count rates for two CCD chips were ∼5 Mn L_α,β_ photons/s and ∼27 000 O K_α_ photons/s, as approximately expected from the Mn:O ratio in the sample (see supplementary material). We find that the Mn spectral region on the CCD also contains background intensity (see the middle panel in Fig. 3), which we attribute to two factors—to the tail of the O K_α_ fluorescence and to x-ray photons scattered from imperfections of the optic surface. However, the prominent O K_α_ fluorescence intensity in the Mn spectral region on the CCD is suppressed by a factor of approximately 300. This enables us to perform Mn L-edge XAS on dilute biological samples such as PS II in solution. Differences corresponding to the “On-Peak” minus the “Off-Peak” signal (i.e., with incident photon energies below 637.5 eV and hence off the Mn L_3_ absorption edge) are also shown in Fig. 3 (bottom). They confirm that the peaks around CCD column number 340 are in fact the Mn L_α,β_ fluorescence signal. We find a ratio of approximately 1:1 for Mn:background signals.

**FIG. 4.**
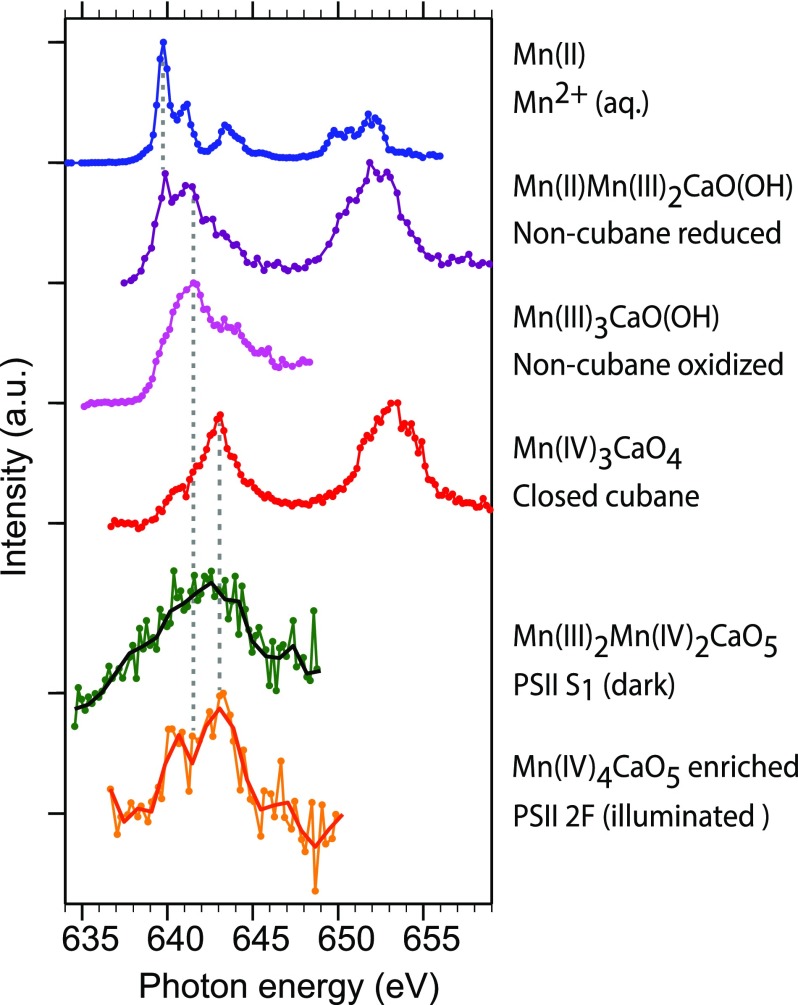
Mn L_3,2_-edge partial-fluorescence yield x-ray absorption spectra of PS II and inorganic high-spin model complexes as measured in solution (see Fig. 1 for molecular structures). Top to bottom: 500 mM Mn^2+^_aq_ solution [the Mn^2+^_aq_ spectrum is the same as in Fig. 2(b)], three inorganic Mn_3_CaO_x_ model complexes with Mn concentrations of 6–15 mM (structures are given in Fig. 1), and the Mn_4_CaO_5_ cluster in PS II with a Mn concentration of 0.8 mM measured for the S_1_ dark resting state (green circles, black line) and an illuminated PS II sample (2 F, meaning that it was illuminated with two optical flashes) in an S_3_-enriched state (orange circles, red line). The solid lines for the PS II measurements are the original data binned to energy regions of 0.8 eV.

For PFY-XAS, the Mn fluorescence signal was integrated, normalized by the total fluorescence signal in the 0th order, and plotted as a function of the incident photon energy (see Fig. 4).

### B. Mn L-edge XAS of dilute molecular complexes in solution

For Mn L-edge XAS scans, the incident photon energy was scanned over the Mn L_3_ or Mn L_2,3_ edges, in steps of 0.13 to 0.3 eV using the SXR beamline monochromator. The photon output of the XFEL is also optimized for each energy step by slightly modulating the electron beam parameters (Vernier scan) to obtain approximately equal incoming photon intensities on the sample over the scan range. At each step, the spectrometer CCD was integrated for equal time spans (5 to 17 s). The Mn L_α,β_ and the O K_α_ signals and the total fluorescence signal in the 0th order reflection were integrated in rectangular regions of interest (ROIs) around the individual Mn L_α,β_ signal spots for each scan step. Focusing the Mn L_α,β_ fluorescence signal with the RZP optics essentially improves the S/N ratio of this signal for dilute biological samples. The incident x-ray flux, focus size, and intensity used for the data collection of each sample are listed in Table I. The choice of these parameters is discussed in Sec. II D and Sec. IV.

**TABLE I.** Samples, x-ray pulse parameters, and estimated influence of x-ray damage mechanisms. c_Mn_ is the Mn concentration, $E_{p}$ is the pulse energy on the sample, $\Delta E_{m}$ is the monochromator bandwidth, $\tau_{p}$ is the pulse duration (FWHM), and *Focus (HxV)* denotes the horizontal and vertical focus sizes (FWHM). ${\bar{n}}_{s}$, ${\bar{\epsilon }}_{s}$, and ${\bar{I}}_{s}$ are the photon fluence, the energy fluence, and the intensity averaged over the probed “skin volume,” i.e., attenuation length times x-ray focus size (FWHM). They are related to the peak values via an averaging factor of $\gamma_{s}$=0.456. Focus sizes with * were measured with a fluence scan imprint method and others on a fluorescent YAG screen. Values with # are based on $E_{p}$ determined from one gas monitor detector (GMD), and all other values are averaged over two GMDs. $D_{s}$ is the x-ray dose absorbed by the probed volume on resonance per pulse. ${\bar{P}}^{m}$ is the average fraction of sequential multi-photon absorption by a molecule with m Mn atoms, and ${\bar{T}}_{NL}$ is the average relative atomic transparency induced by stimulated emission.

Sample	c_Mn_ (mM)	$E_{p}$ (*μ*J)	$\Delta E_{m}$ (eV)	$\tau_{p}$ (fs)	*Focus (HxV)* (*μ*m^2^)	${\bar{n}}_{s}$ (ph/Å^2^)	${\bar{\epsilon }}_{s}$ (J/cm^2^)	${\bar{I}}_{s}$ (TW/cm^2^)	$D_{s}$ (MGy)	${\bar{P}}^{m}$ (%)	${\bar{T}}_{NL}$ (%)
Mn^2+^_aq_	500	4.6	0.4	100	12 × 50*	0.30	0.31	2.9	4.0	1.8	6.5
Mn(II)Mn(III)_2_CaO(OH)	15	9.4^#^	0.6	200	20 × 140	0.13^#^	0.13^#^	0.63^#^	1.7^#^	2.4^#^	0.048^#^
Mn(III)_3_CaO(OH)	10.5	4.6	0.4	100	12 × 60*	0.25	0.25	2.4	3.2	4.4	0.13
Mn(IV)_3_CaO_4_	6	4.6	0.4	100	12 × 60*	0.25	0.25	2.4	3.2	4.4	0.072
PS II (dark)	0.8	3.4	0.4	100	10 × 50*	0.27	0.27	2.6	3.4	6.3	0.010
PS II (2F)	0.8	4.0	0.4	100	10 × 50*	0.32	0.32	3.0	4.0	7.4	0.012

In Fig. 4, we show the Mn L_3_ main absorption spectra of one mononuclear and three multinuclear inorganic Mn complexes with variable electronic and local molecular structures [Fig. 1(b)] (for experimental details including sample preparation, see Sec. IV and supplementary material). Only for the “non-cubane oxidized” compound, the Mn L_3_-edge region is shown (see Sec. IV D).

The data in Fig. 4 demonstrate that the Mn L_3_ peak in the spectra of the Mn_3_CaO_x_ complexes shifts to higher energies with the increasing formal oxidation state of Mn. These spectra were measured at Mn concentrations of 15 mM (Mn(II)Mn(III)_2_CaO(OH), non-cubane, reduced), 10.5 mM (Mn(III)_3_CaO(OH), non-cubane, oxidized), and 6 mM (Mn(IV)_3_CaO_4_, closed cubane). The absolute incident photon energy axis was calibrated with the Mn^2+^_aq_ spectrum (Fig. 4) with reference to the spectra published previously^16^ with an uncertainty of 50 meV. The synthetic Mn_3_CaO_x_ complexes structurally mimic the partial structure of the Mn_4_CaO_5_ cluster of PS II.

**FIG. 5.**
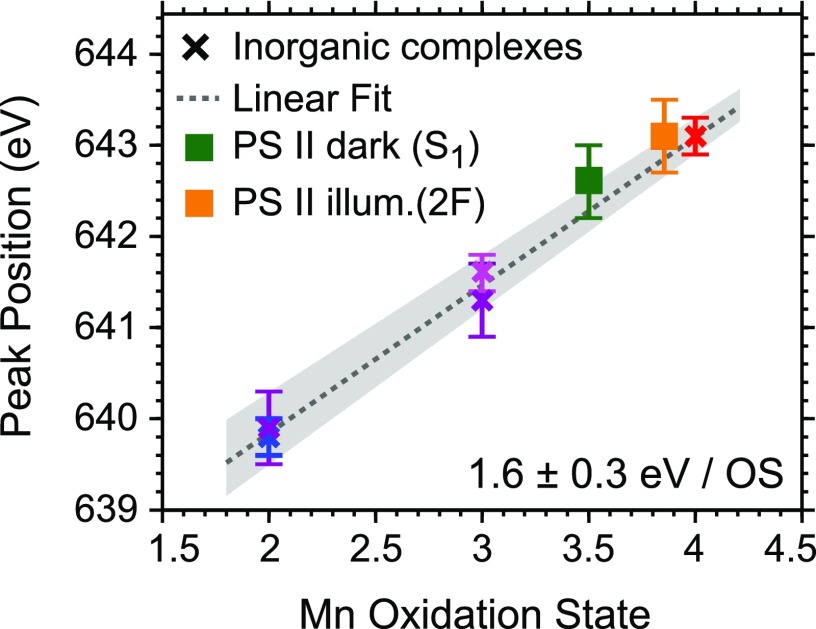
Mn L_3_-edge peak-maximum positions of the spectra shown in Fig. 4 versus formal oxidation states of Mn in the inorganic compounds (the same color code as for the spectra in Fig. 4). The error bars reflect step sizes of  ±1 of the monochromator scan. For comparison, the peak-maximum positions of the Mn L_3_-edge spectra for PS II in the S_1_ (dark) state and the 2 F (illuminated) sample are shown in the expected average oxidation states (error bars of one bin width). Note that the Mn(II)Mn(III)_2_CaO(OH) (non-cubane, reduced) Mn_3_CaO_x_ complex and the PS II samples exhibit mixed oxidation states (see main text), but only for the Mn_3_CaO_x_ complex with two clearly separable Mn L_3_ peaks (see Fig. 4), two peak-maximum positions are given. The gray shaded area reflects the uncertainty of the linear fit.

In a first approximation, we consider the energies for the L_3_ absorption maximum and find that this energy shifts from 639.8 ± 0.2 eV in Mn(II) to 641.6 ± 0.2 eV in Mn(III)_3_ and to 643.1 ± 0.2 eV in Mn(IV)_3_ (where the uncertainties are given by the size of one monochromator step of 0.2 eV). In the Mn(II)Mn(III)_2_CaO(OH) sample with mixed Mn oxidation states, the low-energy peak at 639.9 ± 0.4 eV is assigned to the Mn(II) species, while the high-energy peak at 641.3 ± 0.4 eV corresponds to the Mn(III) species. In Fig. 5, we quantify these observations with a linear fit including all data of the inorganic complexes. We find that the L_3_ maximum shifts by 1.6 ± 0.3 eV per assigned oxidation state of Mn. This is in good agreement with findings based on simple mononuclear Mn complexes.^14^ This analysis, however, neglects the multiplet structure in the spectra.^10^ A more detailed interpretation of our spectra will have to await progress in *ab-initio* theoretical methods^11,39–43^ to correlate the multiplet structures with valence electronic spin and charge densities of the systems.

### C. Mn L-edge XAS of photosystem II

The fifth row of Fig. 4 shows the Mn L_3_ spectrum of the Mn_4_CaO_5_ cluster in PS II in the dark stable (S_1_) state, collected at room temperature from a solution sample with a Mn concentration of 0.8 mM. The spectrum was collected with the same setup and under similar conditions as the three Mn_3_CaO_x_ complexes. Several spectrum scans are averaged, with a total acquisition time of 1.5 h for the entire spectrum or 1.3 min per data point in the spectrum. Due to the low Mn concentration of 0.8 mM in the PS II solution samples, only the L_3_ part of the Mn L-edge was scanned in order to compromise between spectrum statistics and scan time.

As observed in Fig. 4, the Mn L_3_ peak position in PS II is approximately centered between that of the Mn(III)_3_CaO(OH) and the Mn(IV)_3_CaO_4_ complexes, which qualitatively confirms the expected combination of Mn oxidation states (III,III,IV,IV) in the PS II S_1_ (dark resting) state.^1^ This Mn(III)/Mn(IV) mixed oxidation state may also explain the comparably large width of the Mn L_3_ feature of PS II; as the four Mn atoms in the Mn_4_CaO_5_ cluster have Mn(III)/Mn(IV) mixed oxidation states and coordination geometry (six and five coordination sites with bridging oxygen, terminal water, carboxylates, and histidine ligands), such differences likely contribute to the broad Mn L_3_ spectrum of PS II. Moreover, one may speculate on possible spin-spin coupling effects within the Mn_4_CaO_5_ cluster or inhomogeneous broadening effects in the cluster at room temperature, which may additionally broaden the spectrum.

A proof of principle for the experimental feasibility of probing PS II in the successive illuminated states with Mn L-edge XAS is shown in Fig. 4 (bottom row), where a Mn L_3_ spectrum of the PS II 2 F (S_3_-enriched) sample is compared to that of the PS II S_1_ (dark resting) state. Upon successive absorption of visible light photons, PS II advances from the dark stable S_1_ to the S_2_ state and then to the S_3_ state, and it eventually evolves molecular oxygen when advancing from the S_3_ state to S_0_. Each intermediate state is associated with a change in the local charge and spin distributions on the Mn_4_CaO_5_ catalytic site and hence the Mn oxidation states and its geometry.^1^ Experimentally, the protein sample was advanced via a sequence of optical pump laser flashes with well-controlled relative timing, while flowing through the delivery system [green arrows in Fig. 2(a)] before the x-ray pulses probe the sample (see Sec. IV for details). Thus, two flashes (2 F) enrich the PS II sample in the S_3_ intermediate state with a population efficiency of >60%.^44^ The acquisition time of the PS II 2 F spectrum amounts to 1.4 h (1.9 min per data point).

Despite the low S/N ratio of the spectra of PS II, a relative shift of the Mn L_3_ main feature to higher photon energies can be observed for the 2 F-state sample relative to the S_1_-state data. This is consistent with the expected increase in the average Mn oxidation state from 3.5 in the S_1_ state (∼100% Mn(III)_2_Mn(IV)_2_CaO_5_) to an expected value between 3.8 and 3.9 for the PS II 2 F illuminated sample, enriched to ∼60% in the S_3_-state with the Mn(IV)_4_CaO_5_ configuration.^1,30^ The spectral shift is clearly visible in the spectra of PS II in Fig. 4 and amounts to approximately 0.5 eV with a peak position of 642.6 ± 0.4 eV for PS II S_1_ and 643.1 ± 0.4 eV for PS II 2 F (where the size of the error bars is one bin width). These peak positions can now be added to the data in Fig. 5 (square marks), and we find good agreement with the linear increase of 1.6 eV per oxidation state as extracted from the inorganic complexes.

For future time-resolved experiments assessing different time points in the photocycle, better spectral statistics for the PS II samples and progress in the theoretical interpretation^11,39–43^ will be essential, and such effort is underway.

### D. Radiation Damage by intense soft x-ray pulses from XFELs

The possibility to collect spectra from undamaged protein samples at XFELs has been thoroughly demonstrated with x-ray spectroscopy^7,27,45^ and x-ray diffraction^28–30,46–48^ in the hard x-ray regime. However, less experimental evidence has been found in the soft x-ray regime. To ensure that the undamaged sample was probed, we carefully chose the experimental conditions, as we outline in this section. Soft x-ray pulses from the LCLS with photon energies around 640 eV and a pulse duration of ∼100 fs were used for the current experiment. As we demonstrate below, the fs duration of the x-ray pulses guarantees probing of the sample before (dose-dependent) x-ray induced sample damage^19–21^ sets in. On the other hand, since the LCLS x-ray pulses are very intense, they have the potential for undesired spectral effects due to sample damage by sequential multiphoton absorption^49,50^ or x-ray optical nonlinear effects.^51,52^ We have therefore applied experimental protocols, ensuring that these effects do not affect our experimental spectrum (see also Sec. IV and supplementary material). A summary of the experimental sample conditions, x-ray pulse parameters, and estimated influence of the damage mechanisms is given in Table I.

The conventional notion of (dose-dependent) “x-ray damage” addresses the modification of the probed local molecular and electronic structure in metalloproteins and high-valent metal complexes by diffusive radicals and electrons created in the sample bulk after x-ray absorption. It often prevents meaningful experiments of biological systems at synchrotrons even at cryogenic temperature. The classical damage threshold for protein crystallography at synchrotron sources is estimated to be on the order of 30 MGy under cryogenic conditions and on the order of 0.5 MGy at room temperature.^53^ The critical dose for x-ray damage to redox active metal centers, however, was shown to be considerably smaller than the doses relevant to crystallography.^19^ As we have shown above, the features of transition metal L-edge spectra are most sensitive to the formal oxidation state of the probed transition metal. Therefore, the most apparent effect of the diffusive (dose-dependent) x-ray induced sample damage to high-valent Mn complexes in solution is observed by the occurrence of a reduced Mn(II) species in the spectrum.^19^ Similar observations have been made in Ref. 21 for other high-valent transition metal species, which play a crucial role in other metalloenzymes. In Mn L-edge XAS, such damage would be reflected by a comparably sharp peak at ∼640 eV (top row of Fig. 4) which is characteristic of Mn(II). Apparently, neither the spectra of the Mn(III)_3_CaO(OH) and Mn(IV)_3_CaO_4_ samples nor the spectrum of PS II contain any noticeable contribution of a Mn(II) species despite that the dose (here “skin dose” $D_{s}$, see Sec. IV) absorbed by the probed sample volume with each x-ray pulse largely exceeds 0.5 MGy (Table I). This shows that the samples as probed here with the fs soft x-ray pulses from the LCLS XFEL are free from x-ray damage with this dose-dependent diffusive mechanism even at room temperature. This is in agreement with what has been shown as the “probe-before-destroy” concept for metalloproteins in the hard x-ray regime at the LCLS.^7,27^

For the intense x-ray pulses provided by XFELs, on the other hand, we need to consider sequential^49^ and non-sequential nonlinear x-ray optical effects^50–52^ and Coulomb explosion,^54^ which can potentially skew the observed spectrum in an unprecedented manner. The statistical probability P for sequential multi-photon absorption by a Mn complex with m Mn atoms can be denoted with $P^{m}$. We estimate it here using Poisson statistics (see Sec. IV and supplementary material for details) with the measured average photon densities on the sample (Table I) and an absorption cross section σ of 12 Mbarn for the Mn L_3_ resonance.^61^ For the model complexes, we estimate the relative contribution of sequential multi-photon absorption to our spectra $P^{m=3}$ between 2% and 4%, and for the Mn_4_CaO_5_ cluster in PS II, we estimate $P^{m=4}$ between 6% and 7%. These values can be seen as upper limit estimates, as they reflect the case of resonant excitation on the maximum of the L_3_ resonance. The relative effect of nonlinear simulated elastic forward scattering, as confirmed by the parameters given in Table I, is far below 1% for the Mn_3_CaO_x_ molecular complexes and the Mn_4_CaO_5_ cluster in PS II under our experimental conditions. The agreement of our Mn^2+^_aq_ spectrum with spectra of the same sample from a synchrotron source^22^ (see supplementary material) confirms that we do not detect any noticeable spectral distortions due to non-linear effects under our experimental conditions. If this happened, a reduction of the most intense Mn L_3_ peak signal in favor of stimulated x-ray emission in the forward direction would be expected. Last, we note that the energy fluence used in our experiment is ∼10^6^ times lower than that in Ref. 54, and we can hence safely neglect the effect of a Coulomb explosion.

We therefore conclude that the room temperature solution spectra presented here were measured with negligible x-ray damage. The spectrum of the PS II solution sample in Fig. 4, in particular, represents, to the best of our knowledge, the first x-ray damage-free Mn L-edge absorption spectrum of PS II at room temperature. This demonstrates the feasibility of soft x-ray absorption spectroscopy on dilute metalloprotein and inorganic molecular catalysts under functional conditions at XFELs.

## III. CONCLUSION

We herein demonstrate the feasibility of Mn L-edge XAS of dilute high-valent Mn complexes and PS II protein samples in solution with femtosecond soft x-ray pulses from an XFEL. With this method, we can directly probe changes in the unoccupied valence electronic structure concomitant with structural changes and with variations of the valence spin and charge densities. All spectra were measured at room temperature, which is essential for studying the local electronic structure of catalytically active metal centers in (bio)chemical reactions under functioning conditions.

Our results show that under our experimental conditions, the samples were probed without dose-dependent x-ray damage. We also confirm that contributions of non-linear effects such as sequential multi-photon absorption and stimulated emission are negligible at the level of our current experimental conditions and sensitivity. We thereby establish probe-before-destroy soft x-ray absorption spectroscopy of biological samples at XFELs complementary to hard x-ray spectroscopy and diffraction at XFELs. We furthermore set the benchmark for future theoretical approaches to the valence electronic structure of the catalytic site in PS II and in other metalloproteins by reporting experimental L-edge spectra that need to be made accessible to theoretical interpretation. Establishing the sensitivity of L-edge spectra of mono- and multinuclear Mn complexes to the formal oxidation state, the spin state and the valence electronic structure by comparing the experiment and theory are essential for characterizing the Mn-ligand bonds and may enable unique insights into the O-O bond formation mechanism in the water splitting reaction in PS II. We also note that our approach is transferable to a wide range of metalloproteins and molecular inorganic catalysts with 3d transition metals in their catalytic sites and will allow us to directly monitor changes in their electronic structure under catalytically functional conditions in a time resolved manner. The current spectral quality for the PS II samples is limited by the experimental statistics achieved herein. However, the quality of the spectrum for the closed cubane complex, which has a Mn concentration only eight times higher than the PS II sample, points out to what could be achieved with future experiments on dilute samples with longer acquisition times and hence improved spectrum statistics, at existing XFELs. It is important to note that considerably increasing the x-ray pulse energy beyond what was used in the current study does not represent the best solution for improving the spectral quality due to the potential onset of sequential multiphoton absorption and x-ray non-linear effects. Thus, higher repetition rates of the XFEL pulses will greatly enhance the potential of the demonstrated approach. The reported experiments on high-valent inorganic Mn complexes and PS II at room temperature, as well as extensions of our approach to other metalloenzymes and related model compounds in solution, will therefore tremendously benefit from next generation XFEL sources with higher repetition rates such as the European XFEL and LCLS-II.

## IV. MATERIALS/METHODS

### A. Sample preparation and injection

Photosystem II (PS II) was extracted and purified from *Thermosynechococcus elongatus* using the detergent n-dodecyl-β-D-maltoside (βDM)^55^ to a final protein concentration of 70 mg/ml. The purified PS II was resuspended in 100 mM PIPES buffer solution at pH 7 with 5 mM CaCl_2_, 0.015% βDM, and 42% glycerol (w/v) to a final protein concentration of ∼70 mg/ml (=7 mM Chl = 0.8 mM Mn). The Mn(IV) _3_CaO_4_ (closed cubane) model compound sample was prepared as a solution of ∼2 mM LMn(IV)_3_CaO_4_(OAc)_3_(THF) (c(Mn)∼6 mM), the Mn(III)_3_CaO(OH) (non-cubane oxidized) model compound sample was prepared as a solution of 3.5 mM [LMn(III)_3_CaO(OH)(OAc)_2_(OTf)DME]·2OTf (c(Mn)∼10.5 mM), and the Mn(II)Mn(III)_2_CaO(OH) (non-cubane reduced) model compound sample was prepared as a solution of 5 mM [LMn(II)Mn(III)_2_CaO(OH)(OAc)_3_]OTf (c(Mn)∼15 mM). Each sample was synthesized as reported previously^33,34^ and prepared in a 1:1 mixture of anhydrous N,N-dimethylformamide and anhydrous tetrahydrofuran. L denotes thrice deprotonated 1,3,5-tris(2-di(2′-pyridyl) hydroxymethylphenyl) benzene (see supplementary material for further details). The 500 mM solution of solvated Mn^2+^_aq_ was prepared from MnCl_2_·4H_2_O in the 45% glycerol/water mixture (w/v). All samples were loaded into gas tight Hamilton syringes mounted on a KD Scientific syringe pump.

An Electrospinning Microjet^36^ was used to inject the PS II samples, the Mn(II)Mn(III)_2_CaO(OH) (non-cubane reduced) model compound sample, and the Mn^2+^_aq_ solution samples into the liquid jet endstation (LJE)^56^ at vacuum pressures of 10^−4^ to 10^−3^ mbar. The sample syringe was connected to a silica capillary (ID 75 *μ*m, OD 150 *μ*m), coated with polyimide. A charging union at a potential of 3 kV (UH-432, IDEX Health & Science) was inserted into the capillary path, and the counter electrode ∼5 mm below the capillary exit was kept at potentials of −1 kV to −3 kV. The flow rate in the range of 1–3 *μ*l/min was monitored using a flow sensor (Sensirion LG16–0150). For illumination of the PS II sample, three multimode fiber light guides were available, connected to the silica capillary 2, 4, and 6 mm above the x-ray probing region. With two of these, the 2 F state of PS II was prepared with two pulsed laser beams (30 *μ*J, 100 ns, and 420 *μ*m spot size each) from a frequency doubled Nd:YLF laser at 527 nm (Coherent Evolution) and triggered such that each molecule in the specimen was illuminated once per flash (i.e., for a flow rate of 3 *μ*l/min at a rate of 24.1 Hz). In the present setup, the sample takes ∼180 ms between the first and the second flash to complete the conversion of PS II from the S_1_ into the 1 F (S_2_-enriched) state, and between the second flash and the x-ray probe, the sample takes ∼350 ms to complete the conversion into the 2 F (S_3_-enriched) state.^30^ Stated S state enrichments are based on membrane inlet mass spectrometry experiments^57^ reported in Ref. 30.

A Gas Dynamic Virtual Nozzle Jet (GDVN)^35^ was used to inject the Mn(IV)_3_CaO_4_ (closed cubane) and the Mn(III)_3_CaO(OH) (non-cubane oxidized) solution samples at a flow rate of ∼10 *μ*l/min, focused by a He sheath gas jet, previously saturated with the solvent. Fresh sample injection components were used for each sample type in order to avoid contamination effects.

### B. X-ray absorption spectroscopy at the LCLS XFEL

All experiments were performed with the soft x-ray instrument (SXR) of the LCLS XFEL^58^ at a repetition rate of 120 Hz. The x-ray beam was horizontally polarized. The SXR beamline monochromator was tuned to a bandwidth between 0.4 and 0.6 eV (see Table I). For XAS scans, the incident photon energy was varied in steps of 0.13 to 0.3 eV using the SXR beamline monochromator.

The RZP spectrometer used for PFY detection consists of an optical element with effectively 54 x-ray optical reflection zone plate (RZP) structures and two CCD detectors *ANDOR iKon L* covering an effective solid angle of ∼3 × 10^−3^ rad^2^ (3 × 10^−4^ of 4π). For the reduced non-cubane model compound sample, a previous spectrometer version^22,31^ was used, effectively employing 15 RZP structures and one *ANDOR iKon L* detector. The RZP structures have been written on Si wafers and coated with Ni for an improved diffraction efficiency of ∼15%, which in the case of the 54 RZPs was additionally increased to ∼20% in the −1st order for Mn L_α,β_ at ∼637 eV due to a variable profile depth of the zone plate structures. The spectrometer entrance was shielded with a 300 nm (200 nm) Al filter from *LUXEL Corp. (USA)* to block visible and IR light and an additional moveable parylene filter to prevent coating of the Al filter by sample debris during the measurement. Each CCD has 2048 × 2048 pixels with a size of 13.5 × 13.5 *μ*m^2^, binned to 512 × 512 pixels. The active area of a CCD chip covers 27.6 × 27.6 mm^2^. The detection efficiency of the CCD at a photon energy of 640 eV is ∼0.9. The CCD readout noise was ∼5*G CCD counts (rms) at −60 °C (G being the CCD gain factor), whereas a photon energy of ∼640 eV corresponds to ∼30*G CCD counts. For noise reduction, all pixel values below a threshold of 20*G CCD counts were omitted near the Mn spots.

At each scan step, the spectrometer CCD was integrated for equal time spans (5 to 17 s). The Mn L_α,β_ and the O K_α_ signal and the total fluorescence signal in the 0th order reflection are integrated in rectangular regions of interest (ROIs) (drawn around the individual signals) for each scan step, which essentially improves the S/N ratio of the Mn L_α,β_ fluorescence signal. Long-term drifts of the jet position along the axis of the x-ray beam by tens of micrometers on the order of several minutes were considered for the final analysis by a dynamic adaption of the ROIs such that these remained centered on the signal spots on the CCD.

For each spectrum, we measured a PFY-XAS spectrum of the sharp, most prominent Mn L_3_ feature of the Mn^2+^_aq_ solution sample for calibrating the absolute shift relative to the spectrum of Mn^2+^_aq_ solution measured previously.^16^ In addition, a linear stretch factor for the energy axis was fitted for the best agreement of the Mn L_3_ and L_2_ spectral positions of a Mn^2+^_aq_ solution sample to those in our previous work.^16^ We estimate the uncertainty of the energy calibration to be on the order of ∼50 meV.

### C. X-ray induced sample damage

The focus sizes were measured *in situ* with an offline fluence-scan imprint method on lead tungstenate^59^ and a fluorescent YAG screen monitored by an *Infinity K2/SC* microscope in the axis of the x-ray beam. The energy of the x-ray pulses was monitored with gas monitor detectors (GMD) prior to entering the SXR beamline and at the end of the beamline^60^ prior to the focusing optics. The energy of the x-ray pulses on the sample was determined according to the formalism used previously^22^ for both (if available) GMD signals and averaged signals. We estimate 25% absolute uncertainty for the averaged pulse energy values and the deduced magnitudes from the discrepancy of the two GMD values. All estimates of sample damage assume a Gaussian x-ray pulse profile in space and time coordinate. A detailed discussion on our estimates for sample damage is given in the supplementary material

The experimental x-ray pulse characteristics and damage estimates are listed in Table I. The magnitudes ${\bar{n}}_{s}$, ${\bar{\epsilon }}_{s}$, and ${\bar{I}}_{s}$ are averaged over the probed “skin volume,” i.e., attenuation length times x-ray focus size (FWHM), and are related to the peak values via an averaging factor of $\gamma_{s}$=0.456 (see supplementary material for details). The “skin doses” $D_{s}=(0.402{\cdot E}_{p})/(\rho \Lambda \cdot V\times H)$ absorbed by the probed “skin volume” (focus size V×H (FWHM) times x-ray attenuation length Λ∼ 0.8 *μ*m) were calculated with the pulse energy $E_{p}$ and the average sample density of ρ∼ 1 g/cm^3^ (for H_2_O as a solvent). The skin-volume averaged probability for multi-photon absorption ${\bar{P}}^{m}$ on the Mn L_3_ resonance by a molecule with m Mn atoms was calculated via ${\bar{P}}^{m}\left(\mu \right)=1-P_{\mu \;}\left(1\right)-P_{\mu }\left(0\right)/1-P_{\mu }\left(0\right),$ where $P_{\mu \;}\left(k\right)=\mu^{k}\exp\left(-m\cdot \mu \right)/k!$ is the discrete Poisson distribution and $\mu =\mu_{res}={\bar{n}}_{s}\cdot \sigma_{res}$ is the resonant atomic absorption probability for a single photon with the skin-volume averaged area density of photons ${\bar{n}}_{s}$ and an absorption cross section of $\sigma_{res}=$ 12 Mbarn^61^ on the Mn L_3_ peak resonance of aqueous Mn(II) ions. The skin-volume averaged transparency, induced by stimulated emission, ${\bar{T}}_{NL}\approx 2{\tilde{\rho }}_{22}\left(\infty \right)$, is estimated with ${\tilde{\rho }}_{22}\left(\infty \right)$ from the study by Stöhr and Scherz^51^ using the experimental Mn concentrations and averaged intensity ${\bar{I}}_{s}$ from Table I, the Mn 2p life time width of Γ=0.32 eV,^13^ and a dipole transition width $\Gamma_{x}=0.93\;$ meV. For consistency, $\Gamma_{x}$ is deduced from $\sigma_{res}$ as stated above via the relation ${2.9\times \sigma }_{res}={\lambda_{0}^{2}\Gamma }_{x}/(\Gamma \pi )$, where the empirical factor of 2.9 (Ref. 51) relates the theoretical to the experimental cross section on the L-edge resonance.

### D. Data selection and analysis

The projected CCD signals in the middle row of Fig. 3 show integrated photons per second, summed along the vertical CCD columns in the top row. Prior to calculating the difference in the “On-peak” minus “Off-peak” data (bottom row of the figure), the latter was normalized to the former for equal counts in the 0th order TFY signal (sum of counts in CCD columns 65 to 285).

For better visibility, in Fig. 4, the background level of each spectrum, averaged from all spectrum points with hν≤ 637.5 eV, was subtracted from each spectrum. The spectra were then normalized to their maximum value.

Data points for which the required stability of the liquid sample delivery could not be assured were omitted. For this reason, only the Mn L_3_ edge region is shown for the “non-cubane oxidized” compound (center row of Fig. 4), and the Mn L_2_ edge data were omitted.

For the low concentrated PS II samples, only PFY-XAS scans with clearly identifiable Mn PFY signal spots on the CCD were selected and averaged for the final data set. For PS II in the dark state (S_1_), two spectrum scans were averaged, where the spectral intensities were weighted by the “On-peak” Mn L fluorescence counts (summed fluorescence counts in the Mn L_3_ absorption peak (FWHM)) and normalized to the averaged background level (counts on the low energy side of the Mn L_3_ feature).

## V. SUPPLEMENTARY MATERIAL

See supplementary material for further details on the sample preparation, count rate estimates, and the detailed parametrization of the x-ray pulses and x-ray damage.
